# Filling gaps in models simulating carbon storage in agricultural soils: the role of cereal stubbles

**DOI:** 10.1038/s41598-021-97744-z

**Published:** 2021-09-15

**Authors:** Arezoo Taghizadeh-Toosi, Bent T. Christensen

**Affiliations:** 1 Department of Agroecology, AU-Foulum, Aarhus University, 8830 Tjele, Denmark; 2 Danish Technological Inistitute, DTI, Agro Food Park 15, Skejby, 8200 Aarhus N, Denmark

**Keywords:** Environmental impact, Carbon cycle

## Abstract

Carbon (C) input is a prerequisite for the formation of soil organic matter and thus for soil organic C (SOC) sequestration. Here we used the C-TOOL model to simulate SOC changes in a long-term field experiment (1932–2020) at Askov, Denmark, which involved four different levels of nutrients added in mineral fertilizer (0, 0.5, 1, 1.5 NPK) and a four-crop rotation. The C input into soils consists of belowground and aboveground plant biomass and was estimated using allometric functions. The simulation showed that modelled SOC based on standard allometric functions of C input from crop residues did not adequately matched measured SOC contents. However, applying modified allometric functions based on current and the previously measured results for aboveground and belowground C inputs in winter wheat and grass clover in rotations provided much better match between simulated and measured SOC contents for fertilized treatments at normal and high level of fertilization. This improved indicators of C-TOOL model performance (e.g. yielding RMSE of 2.24 t C ha^−1^ and model efficiency of 0.73 in 1.5 NPK treatment). The results highlight that standard allometric functions greatly overestimates the amount of C in winter wheat stubble left after harvest in treatments dressed with NPK compared with modified functions. The results also highlight further needs for improvement of allometric functions used in simulation models for C-accounting in agroecosystems.

## Introduction

The global pool of soil organic carbon (SOC) down to a depth of 1 m contains about twice as much C as the atmosphere whereby changes in SOC storage impact levels of atmospheric carbon dioxide (CO_2_)^1^. Changes in SOC due to changes in land use and soil management occur slowly and against a large background of SOC with high spatial variability^2,3^. Thereby, mathematical simulation models that accommodate long periods and complex interactions among climatic, soil and management factors become essential tools to predict long-term developments in SOC storage on national and regional scales. To verify the reliability of models simulating management impact on SOC storage, long-term field experiments provide essential data sets against which the performance of simulation models can be tested^4,5^.

Essentially, the storage of SOC in ecosystems reflects the balance between C input through photosynthesis (primary production) and/or management, and C loss via decomposition. Net primary production (NPP) is a summary measure of aboveground and belowground plant biomass and provide a basis for establishing the C inputs in SOC simulation models. To estimate the fraction of NPP that contributes C inputs to the soil, dedicated SOC simulation models apply allometric scaling that aims to reflect the relationships among different plant fractions^5,6^.

Allometric functions adopted for agroecosystems may be established empirically but are more often based on simple equations with C inputs being related linearly or non-linearly to the harvested fraction of the crop. However, the choice of allometric function represents a most critical step in modelling developments in SOC storage, and allometric relationships need further refinement from empirical data. Often this aspect is neglected when using SOC simulation models. It is recognized that allocation patterns are under genetic control^7,8^ as illustrated by dwarfing genes that over time have decreased dry matter allocation to stems and increased allocation to grains in cereals^9^. In addition, aboveground crop biomass responds more to addition of nutrients than belowground plant fractions^29^. Allometric functions based on fixed root to shoot ratio links to the ideas of functional equilibration between root and shoot^10^. It is assumed that root growth is limited by assimilate supply from the shoot and shoot growth by nutrient supply from the root. However, recent studies involving temperate crops provide evidence that seriously question the realism of conventional allometric functions based on the concept of functional equilibrium between roots and shoot^11–15^. Establishing allometric functions based on measurements remains among the unresolved gaps associated with the use of SOC simulation models.

In cereal intensive cropping system based on mineral fertilizers, incorporation of crop residues following grain harvest represents an essential C input to the soil. However, cereal straw has become an important source of bioenergy for power plants and for 2nd generation biofuels with an expected increase in straw removal along with implementation of plans to phase out fossil energy sources. Conventional combiner harvest of cereals with subsequent removal of straw by baling may leave a substantial fraction of aboveground crop residues (including stubbles and chaff) in the field for subsequent incorporation^16–18^. When using conventional harvest indices and standard allometric functions based on grain yields only, simulation of changes in SOC storage as affected by removing straw by baling may be misleading. This calls for information on the soil C input from crop residues left after grain harvest and the distribution of harvest residues among different fractions as affected by different yield levels. Most important residue fractions left after grain and straw removal include stubbles and chaff.

In this paper, we combine a study on the distribution of aboveground fractions of winter wheat with previous modifications of standard allometric functions in order to reduce the uncertainty linked to C input estimation and improve the estimation of SOC changes using simulation models. We focus on uncertainties associated with stubble part expected to overestimate C input in SOC models using standard allometric functions to simulate effects of straw removal. We then tested the modified model set-up against data for winter wheat grown with four different rates of mineral fertilizers in the Askov Long-term Experiment (Askov-LTE) during 1932 to 2020.

## Materials and methods

### The Askov-LTE: experimental design and data source

This study uses data from the Askov Long-Term Experiment (Askov-LTE)^19^, established in 1894 at the Lermarken site of Askov Experimental Station, South Jutland (55° 28′ N, 09° 07′ E). The soil is a coarse sandy loam with 11% clay and an average bulk density of 1.55 g cm^−3^ and classifies as Typical Hapludalf (USDA Soil Taxonomy). Since 1923, sampling of soil from the 0–20 cm layer has occurred every four years, with soils archived in dry condition and later analyzed for C content using dry combustion methods. Table 1 shows average annual temperature and precipitation during the period 1932–2020. The Askov-LTE grows a four-course crop rotation of winter cereals, row crops, spring cereals and grass-legume mixtures (Table 1) and includes four replicate blocks (termed B2-, B3-, B4- and B5-field). Each field is with replicated treatments of nutrients applied at different rates in animal manure or in mineral fertilizers. Annual applications of mineral fertilizers occur in early spring (March or April). Mineral fertilizer N was calcium nitrate until 1973 when replaced by ammonium nitrate. Superphosphate was the P source until 2006 followed by triple superphosphate. For most years, the source of K was potassium chloride. Cereal harvests remove aboveground crop residues, leaving ~ 5–10 cm stubbles. For root crops, the harvest procedure removes roots as well as top. Silage maize (replacing root crops in 2006) and grass-clover crops are whole-crop harvested. Field management, other than experimental nutrient treatments, has followed the general development in conventional Danish agriculture. That includes crop varieties, tillage, crop protection and liming, crop planting and harvest procedures.

**Table 1:** Average annual temperature and precipitation during 1932–2020, crops included in the four-course rotation, and amounts of nutrients added in the 1 NPK (mineral fertilizer) treatment. Data from Christensen et al. (2019)^19^ and Macholdt et al. (2021)^20^.

Period	Average annual temperature (^o^C)	Average annual precipitation (mm)	Crop rotation element	1 NPK (kg ha^−1^)		
				N	P	K
1932–1948	8.0	641	Winter wheat	68	14	57
			Root crops	160	25	108
			Spring cereals	50	15	58
			Grass clover	0	15	58
1949–1972	7.7	698	Winter wheat	70	16	66
			Root crops	160	38	100
			Spring barley	50	16	33
			Grass clover	0	0	66
1973–2005	8.1	720	Winter wheat	100	19	88
			Root crops	225	44	196
			Spring barley	75	14	65
			Grass clover	0	0	0
2006–2020	8.9	895	Winter wheat	150	30	120
			Silage maize	150	30	120
			Spring barley	100	20	80
			Grass clover	0	0	0

For the present study, yields and soil C data were obtained in the B2e-field (which is east part of B2 field) from treatments exposed to different rates of mineral fertilizers (0 (unmanured), 0.5, 1, and 1.5 NPK; see Table 1). Each treatment comes with four replicates. Crop harvest yields and soil C contents were from the database associated with the Lermarken experiment^19^.

### Determination of aboveground wheat residue fractions

To examine the importance of accounting for cereal stubbles in modelling soil C levels following removal of harvestable straw, a separate study was performed in 2020 in the B2e-field. This year the B2e-field grew winter wheat (cv. Sheriff, sown 19-09-2019). In the central part of each of the four plot replicates of the four mineral fertilizer rates, mature wheat plants within one m^2^ was cut manually at ground level and subsequently separated into grain, chaff, and straw including stubbles. A plot combiner harvested another eight m^2^ within the same plot for separation of the fractions grain, chaff, and straw without stubbles. Subtracting the fraction straw without stubbles from the fraction straw including stubbles allowed us to quantify the stubbles fraction. The plot combiner then harvested the remaining part of the plot following the standard procedure used for harvest of cereals in the Askov-LTE. This provided two main fractions of grain and straw + chaff. These fractions allow comparison with previous yield data stored in the Askov-LTE database. Analysis for C content in winter wheat fractions were performed by dry combustion.

### C-TOOL modelling

The C-TOOL model is a dedicated SOC model, simulating medium to long-term changes in soil C storage in agricultural mineral soils. C-TOOL was developed and calibrated for arable land and grassland in Denmark^21–23^. The model includes three conceptual pools (FOM: fresh organic matter; half-life of 0.5 year, HUM: humified organic matter; half-life of 20 years, and ROM: resistant organic matter; half-life of 1500 years) in both topsoil (0–25 cm) and subsoil (25– 100 cm). Driving variables are soil texture (clay content), soil temperature, soil C-to-N ratio, and the type, quantity and application date of organic inputs. The model assumes air temperature to be the overarching climatic driver for C turnover in soil. The initialization of C-TOOL applied C pool sizes from simulations based on measured data (1986, 1997 and 2009) from the Danish nationwide C monitoring network^21–23^.

Like most other models simulating C turnover in soil, C-TOOL use standard allometric functions to calculate soil C inputs from above- and below-ground plant residues. In standard allometric functions, the soil C input from crops relies the amount of C harvested in the main product (e.g. cereal grains). In current study, standard allometric functions relate primary harvestable crop yields, obtained during 1932 to 2020, to total aboveground and belowground production. Further, we apply modified allometric functions, introduced in previous studies^14,15^ to establish improved estimates of belowground C inputs for winter wheat and grass-legume crops. In these modified allometric function, the belowground C inputs from winter wheat and grass legume mixture relied on measurements and allometric functions modified accordingly. Now we add one further modification based on the data from the current study of winter wheat aboveground fractions. This allows for better estimates of the distribution of aboveground biomass on grain, straw, chaff and stubbles fractions following combiner harvest. The estimation on total amount of stubbles as affected by different fertilization levels adds one further modification to the previously introduced modifications of standard allometrics. Supplementary materials provide all equations and parameters.

### Model validation

To evaluate model performance, we compared measured and simulated soil C stocks using MATLAB (MathWorks Inc., 2016). The difference between simulated and measured values was calculated as the root mean square error, RMSE and the model efficiency (EF)^24^.

$$ RMSE = \sqrt {\frac{{{\Sigma }_{i = 1} (P_{i} - O_{i} )^{2} }}{n}} $$

where $O_{i}$ is the measured value, $P_{i}$ is the simulated value and $n$ is the number of paired values.

$$ EF = \frac{{\mathop \sum \nolimits_{i = 1}^{n} (O_{i} - \overline{O})^{2} - \mathop \sum \nolimits_{i = 1}^{n} (P_{i} - O_{i} )^{2} }}{{\mathop \sum \nolimits_{i = 1}^{n} (O_{i} - \overline{O})^{2} }} $$

where $\overline{O}$ is the mean of measured values.

## Results

**Figure 1:**
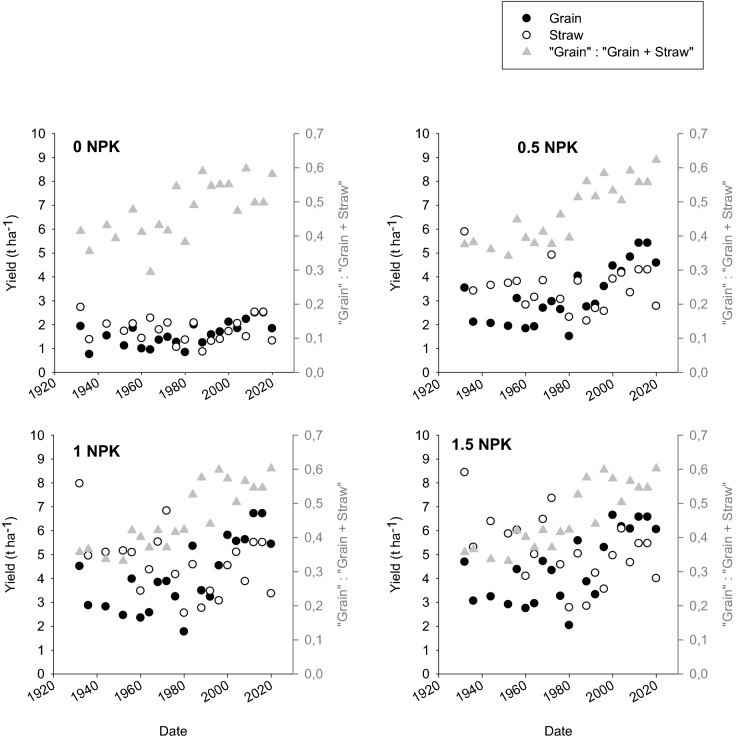
Grain and straw yields for winter wheat obtained in Lermarken during 1932 to 2020 on unfertilised plots (0) and plots treated with different rates of mineral fertilizers (NPK). The ratio between grain and grain + straw is shown on right y axis. Straw includes chaff but not stubbles.

Figure 1 shows yields of winter wheat grain and straw obtained in the B2-field during 1932 to 2020. Data extracted from the Askov-LTE database reports yields with 85% dry matter. The ratio of “grain” to “grain + straw” relies on harvested fractions and straw includes chaff but not stubbles.

**Figure 2:**
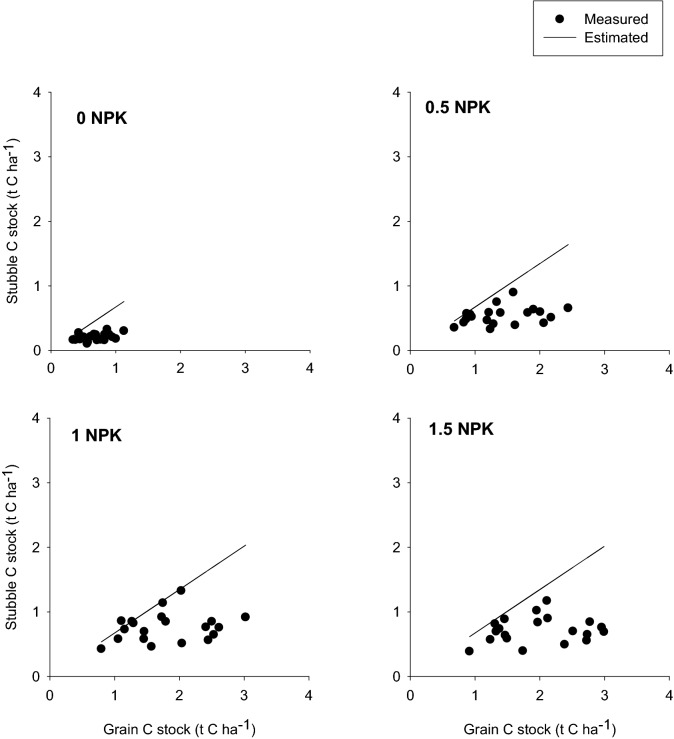
The relationship between grain C stock measured during 1932–2020 and stubble C stock based on measurements in 2020 (circles; see Table 2). Stubble C stock calculated with standard allometric functions for period 1932–2020 (solid line).

Table 2 shows the distribution of C among winter wheat aboveground fractions obtained in 2020 in B2e-field. Subsequently, we applied the measured relationship obtained in 2020 between grain yield and stubbles to grain yield data for the period 1932–2020. The modelled amount of stubble C content relied on standard allometric functions. Figure 2 shows the relationship between the amount of C in stubbles (both measured and modelled) and the measured amount of C in grain yields for the period 1932–2020.

**Table 2:** The amounts of C in mature winter wheat grain, straw (without stubbles), chaff, and stubbles fractions measured in the B2e-field in 2020. Average ± standard error of the mean (SE, n = 4).

Treatmen t (NPK)	Grain (t C ha^-1^)	Straw (t C ha^−1^)	Chaff (t C ha^−1^)	Straw + Chaff (t C ha^−1^)	Stubbles (t C ha^−1^)
0	0.91 ± 0.06	0.54 ± 0.20	0.11 ± 0.01	0.66	0.17 ± 0.35
0.5	2.27 ± 0.14	1.09 ± 0.42	0.28 ± 0.00	1.38	0.45 ± 0.16
1	2.69 ± 0.15	1.32 ± 0.55	0.35 ± 0.03	1.67	0.60 ± 0.38
1.5	3.00 ± 0.36	1.62 ± 0.52	0.36 ± 0.03	1.98	0.60 ± 0.48

**Figure 3:**
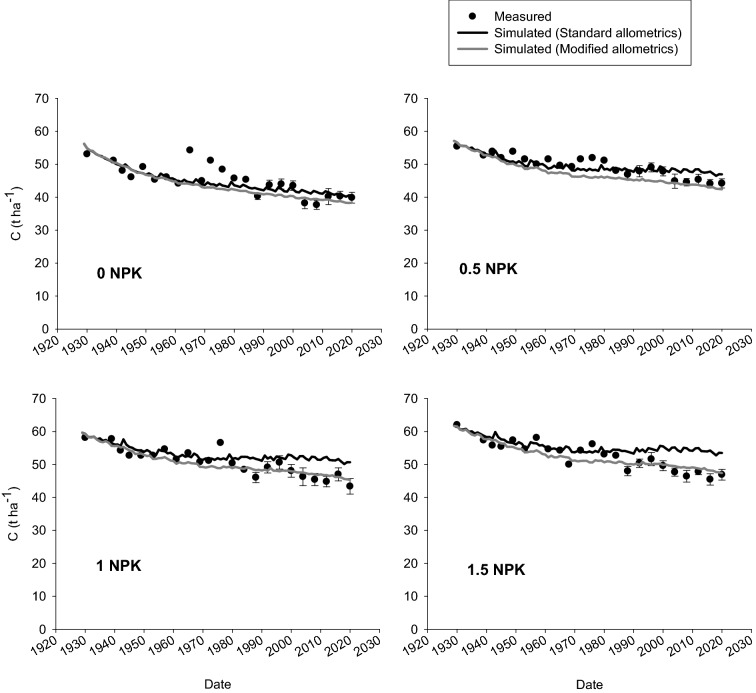
Measured (circles) and simulated (lines) soil C stock (t C ha^−1^) in B2e-field topsoil (0-25 cm) of treatments exposed to 0, 0.5 NPK, 1 NPK, and 1.5 NPK during 1923–2020. Error bars are standard error of the means (SE).

The modified allometric approach involved changes in allometric functions for winter wheat and grass-clover crops. Figure 3 shows the measured and simulated values for C contents in the B2-field topsoil during 1932–2020 and Table 3 shows the statistical analyses on model performance. The RMSE values decreased and EF increased by increasing the fertilization rate, emphasizing the lack of a fixed relationship between winter wheat grain yield and the amount of C in stubbles.

**Table 3:** Values of root mean square error (RMSE, t C ha^−1^) and model efficiency (EF) for simulated top SOC of each treatment.

Treatment	Allometric functions	RMSE*	EF^§^
0 NPK	Standard	3.20	0.50
	Modified	3.56	0.38
0.5 NPK	Standard	2.30	0.5
	Modified	2.95	0.18
1 NPK	Standard	3.84	0.09
	Modified	2.28	0.68
1.5 NPK	Standard	4.39	−0.05
	Modified	2.24	0.73

*RMSE values can range from 0 (representing complete match) to + ∞.

^§^Values for EF range from -∞ to 1 (representing perfect model). A positive EF value indicates that the simulated values describe the variation in the measured data better than the mean of the observations. A negative value indicates that the simulated values describe the data less well than a mean of the observations.

## Discussions

The design of the plot combiner used for cereal harvest in the Askov-LTE ensures collection straw and chaff on a weighing tray mounted on the back of the combiner. This allows quantification and then removal of the straw and chaff fractions from the plots. Thereby, the aboveground crop residue fraction of cereals grown in the Askov-LTE relates to stubbles and allows us to isolate the effect of this fraction in the present study. Conventional combining with subsequent removal of straw by baling leaves not only stubbles but also a fraction of the straw and most of the chaff in the field. The straw and chaff fractions escaping baling provide an additional C input, often neglected when modelling effects on SOC storage of straw removal on regional and national scales.

In conventional cropping systems relying on mineral fertilizers, biomass production is dominant source of C input to the soil compartment of agroecosystems. Better understanding of all components of the C cycle will provide answers to some problems of C turnover in agricultural lands particularly that concerned with biomass removal for energy and maintenance of a desirable level of soil organic matter. Despite the key role of crop residues in C cycle, there are few quantitative measurements of residue fractions returned to soil, especially at national scales. To better quantify the importance of cereal residues left after harvest, we relied on field measurements of winter wheat C stubbles to improve C input estimates for modelling. Our results demonstrate that standard allometrics used in most simulation models will overestimate the C input provided by leaving stubbles in the field.

There has been an increase in global cereal production, and also the change from long-strawed variety with a modern short-strawed variety^25–27^. Since wheat was introduced in the Askov-LTE in 1932, there has been an increase in global cereal production, and also the change from long-strawed variety with a modern short-strawed variety^26,27^ . In addition, the ratio of grain to straw varies and depends on a range of factors, including the crop type, variety, nutrient supply and seasonal conditions. While the C assimilated in the harvestable aboveground biomass is removed from the field in the form of grain and straw, the C added to soil as stubble, chaff, root biomass and rhizodeposition is retained and add to soil C storage. Generally, it is considered for every 1t ha^−1^ of grain yield there will be 1.5–2 t ha^−1^ of cereal stubble remaining immediately after harvest^28^. The results from the current long-term study with different mineral fertilizer levels (1932–2020) show that the winter wheat grain yields increase from around 1 t ha^−1^ in the unmanured treatment (0 NPK) to 7 t ha^−1^ in the 1.5 NPK treatment. However, the 2020 measured and the 1932–2019 estimated input of C with wheat stubble remains almost constant regardless of fertilizer rate (excluding the long-term unmanured treatment) and accounts for 1 t C ha^−1^. This estimate is only half of that modelled when allometric relations did not rely on measurements of stubbles left as aboveground crop residues. Therefore, our results suggest that most current SOC models may overestimate this C input, and that the accuracy of aboveground biomass estimation still falls behind what is required for simulation models involved in regional and national C accounting.

Measurements of soil C contents in all four fields of the Askov-LTE show an on-going slight decline in SOC storage regardless of fertilization level^19^. This includes the B2e-field used in the present study (Fig. 3) while simulations based on standard allometrics suggest an almost steady state situation. This probably ascribes to the nature of simple standard allometrics originally developed and based on even fewer studies than are currently available. In model using standard allometric relationships, crop C inputs to soil are estimated using yield data and fixed coefficients for stubble, roots and rhizodeposition production^29,30^. Therefore, considering only fixed coefficients would overestimate residue carbon input at high yields levels, either from overestimation of stubble or belowground part.

Our results highlight the needs for further improvement of input estimation in models simulating SOC turnover, including inputs from other crops with different leftover crop residues and management systems. The modification on allometric functions of stubble C input presented here appears to give a more realistic estimation of SOC changes than previous estimates.

## Conclusions

Three primary conclusions may be drawn from this study: (1) the aboveground residues left in the form of wheat stubbles is a smaller component of the soil C input than assumed by standard allometric assumptions at normal and high fertilization rate, (2) the modification of allometric functions based on actual measurements of aboveground fractions resulted in a lower C input estimate from winter wheat than previously reported, and (3) new allometric modification on under-represented parts of aboveground crop residues will improve regional and national scale accounts of SOC stored in agricultural land with normal and high level of fertilization. These conclusions, in addition to the general approach outlined in this study, may be useful for reporting soil C accounts in a wider international context.

## Supplementary Information

Supplementary Information.
