# Supplementary material for: Filling gaps in models simulating carbon storage in agricultural soils: the role of cereal stubbles
Source: Sci Rep. 2021 Sep 15;11:18299. doi: 10.1038/s41598-021-97744-z (PMC8443642; doi:10.1038/s41598-021-97744-z)
Supplement: Supplementary file 1 — Supplementary Information. [file 41598_2021_97744_MOESM1_ESM.docx]

**Supplementary Materials**

**Filling gaps in models simulating carbon storage in agricultural soils: the role of cereal stubbles**

Arezoo Taghizadeh-Toosi *^1,2^* & Bent T. Christensen *^1^*

*^1^* *Aarhus University, Department of Agroecology, AU-Foulum, DK-8830 Tjele, Denmark*

*^2^ Danish Technological Inistitute, DTI, Agro Food Park 15, Skejby 8200, Aarhus N, Denmark*

*Standard allometric functions*

Taghizadeh-Toosi et al. (2014a) detail the standard allometric function for converting measured crop yields to soil C inputs in C-TOOL. The soil C input from crops is calculated using the amount of C ($C_{P})$ in dry matter yield ($Y_{DM})$of the main product ($C_{P})$ as:

$$C_{P}=Y_{DM}CC Equation (1)$$

where *CC* = 0.45 is the C concentration in the harvested product.

The aboveground C in crop residues, *C_S_*, depends on the harvest index, *HI* (Table 1). If the secondary product (i.e. straw) is not harvested:

$$C_{S}=\left( 1/HI-1 \right)C_{p} Equation (2)$$

If the secondary product is harvested, then:

$$C_{S}=\left( 1/HI-1-F_{S}F_{SH} \right)C_{P} Equation (3)$$

where *F_S_* is the biomass of the secondary product as a proportion of the main product yield and *F_SH_* is the proportion of the secondary product that is harvested.

Belowground C input from roots and rhizodeposition, *C_iRE_*, is calculated as:

$$C_{iRE}=F_{RE}/\left( \left( 1-F_{RE} \right)HI \right)C_{P} Equation (4)$$

where *F_RE_* is root and rhizodeposition C (below-ground C) as a proportion of total C assimilation (Table 1). The proportion of belowground C deposited in the topsoil of the C-TOOL model (0-25 cm) was estimated to be 0.7 for winter crops, and 0.9 for grasslands. In the current study, this approach is termed standard allometric functions.

**Table 1.** Coefficients for allometric function in C-TOOL for selected crops. Harvest index of main crop relative to aboveground biomass (HI), biomass of secondary crop product as proportion of yield of the main crop product (*F*_S_), root biomass and rhizodeposition as proportion of total C assimilation (*F*_RE_) (Taghizadeh-Toosi et al*.*, 2014a; Taghizadeh-Toosi & Olesen, 2016). *Note:* The labelling of the variables differs from that used in the original publication of the C-TOOL model (Taghizadeh-Toosi et al., 2014a) but align with that adopted by Keel et al. (2017). In the original version *C*_P_ refer to *C*_main_, *Y*_DM_ to *Y*_main_, *CC* to *ε,* HI to *α*, *C*_S_ to *C*_resid_, *C*_iRE_ to *C*_below_, *F*s to *δ, F*_SH_ to *ζ*, *F_RE_* to *β*, *C*_top_ to *C*_rootTop_, *C_s_*_ub_ to *C*_rootSub_, and *F*_top_ to *ξ*.

| Crop | HI | *F*_S_ | *F*_RE_ |
| --- | --- | --- | --- |
| Winter wheat | 0.45 | 0.55 | 0.25 |
| Spring barley | 0.45 | 0.55 | 0.17 |
| Winter barley | 0.39 | 0.55 | 0.17 |
| Rye | 0.38 | 0.80 | 0.25 |
| Oat | 0.40 | 0.60 | 0.17 |
| Grass and grass-clover | 0.70 | 0 | 0.45 |
| Potatoes | 0.70 | 0 | 0.11 |
| Fodder beets | 0.70 | 0.34 | 0.12 |
| Maize for silage | 0.85 | 0 | 0.15 |

*Modified allometric functions*

We applied a modified allometric functions for grass ley and winter wheat by Taghizadeh-Toosi et al. (2016b) and Taghizadeh-Toosi et al. (2020).

For grass-legume ley, the modification relied on root C inputs measured by Cong et al. (2019) in the Askov long-term experiment after the second cut in late summer. In that experiment, the root biomass, measured by coring and soil-washing techniques, and was independent of fertilization regimes. Here, we used root C (1.4 t C ha^-1^) determined for grass-legume ley with standard rates of mineral fertilizers (1 NPK treatment, see section 2.4.1 below) and applied the net rhizodeposition/root factor (0.5) reported for grass by Pausch and Kuzyakov (2018). Thereby, the net rhizodeposition became 0.5 × 1.4 = 0.7 t C ha^-1^ while the total belowground C input became 1.4 + 0.7 = 2.1 t C ha^-1^. For all treatments and experiments, we applied a harvest index (HI) of 0.8 for grass ley to estimate C inputs from aboveground biomass residues. The HI for other crops is as in Table 1. The aboveground C assimilation, based on C in harvested herbage (4.8 T C ha^-1^) reported by Cong et al. (2019), became 4.8 / 0.8 = 6.0 t C ha^-1^ and total C assimilation 6.0 + 2.1 = 8.1 t C ha^-1^. Thus, *F*_RE_ (the fraction of total C assimilation allocated to root biomass and net rhizodeposition) changed from 0.45 in the standard allometric function (Table 1) to 2.1/8.1 = 0.26 in the modified allometric approach.

For winter wheat, similar approach was used using measured data in Askov 2020 and from treatments with standard fertilization level in the Broadbalk Winter Wheat Experiment. Harvest index based on the level of fertilization and measured left aboveground residues after harvest in 2020; changed from 0.45 to 0.4–0.6. The amount of C input from root was set to 1.3 t C ha^-1^; independent of fertilization rates.

**References**

Keel, S. G., Leifeld, J., Mayer, J., Taghizadeh-Toosi, A. & Olesen, J. E. Large uncertainty in soil carbon modelling related to carbon input calculation method. *European Journal of Soil Science* **68**, 953-963 (2017).

Taghizadeh–Toosi, A. *et al.* C-TOOL: a simple model for simulating whole-profile carbon storage in temperate agricultural soils. *Ecological Modelling* **292**, 11-25 (2014a).

Taghizadeh-Toosi, A. *et al.* Changes in carbon stocks of Danish agricultural mineral soils between 1986–2009. *European Journal of Soil Science* **65**, 730-740 (2014b).

Taghizadeh-Toosi, A. & Olesen, J. E. Modelling soil organic carbon in Danish agricultural soils suggests low potential for future carbon sequestration. *Agricultural Systems* **145**, 83-89 (2016a).

Taghizadeh-Toosi, A., Christensen, B. T., Glendining, M. & Olesen, J. E. Consolidating soil carbon turnover models by improved estimates of belowground carbon input. *Nature Scientific Reports*, doi:10.1038/srep32568 (2016b).

Taghizadeh-Toosi, A. *et al.* Visiting dark sides of model simulation of carbon stocks in European temperate agricultural soils: allometric function and model initialization. *Plant & Soil* **450**, 255-272 (2020).
